# Myc regulates programmed cell death and radial glia dedifferentiation after neural injury in an echinoderm

**DOI:** 10.1186/s12861-015-0071-z

**Published:** 2015-05-30

**Authors:** Vladimir S Mashanov, Olga R Zueva, José E García-Arrarás

**Affiliations:** University of Puerto Rico, Rio Piedras, PO Box 70377, San Juan,, PR 00936-8377 USA

**Keywords:** Myc, Regeneration, Central nervous system, Dedifferentiation, Cell death

## Abstract

**Background:**

Adult echinoderms can completely regenerate major parts of their central nervous system even after severe injuries. Even though this capacity has long been known, the molecular mechanisms that drive fast and complete regeneration in these animals have remained uninvestigated. The major obstacle for understanding these molecular pathways has been the lack of functional genomic studies on regenerating adult echinoderms.

**Results:**

Here, we employ RNA interference-mediated gene knockdown to characterize the role of *Myc* during the early (first 48 hours) post-injury response in the radial nerve cord of the sea cucumber *Holothuria glaberrima*. Our previous experiments identified *Myc* as the only pluripotency-associated factor, whose expression significantly increased in the wounded CNS. The specific function(s) of this gene, however, remained unknown. Here we demonstrate that knockdown of *Myc* inhibits dedifferentiation of radial glia and programmed cell death, the two most prominent cellular events that take place in the regenerating sea cucumber nervous system shortly after injury.

**Conclusions:**

In this study, we show that *Myc* overexpression is required for proper dedifferentiation of radial glial cells and for triggering the programmed cell death in the vicinity of the injury. *Myc* is thus the first transcription factor, whose functional role has been experimentally established in echinoderm regeneration.

**Electronic supplementary material:**

The online version of this article (doi:10.1186/s12861-015-0071-z) contains supplementary material, which is available to authorized users.

## Background

Injuries to the central nervous system (CNS) in mammals result in devastating consequences because of the limited capacity of the lesioned neurons to regenerate across the wound site and re-establish appropriate connections. On the other hand, there are animals who can completely regenerate their severed CNS in a fast and efficient way. Among these animals, echinoderms are of particular interest, because their phylogenetic position among basal deuterostomes makes them equally suitable for studies of evolution of neural regeneration in the animal kingdom and for looking for insights into how CNS regeneration can be improved in mammals.

**Fig. 1:**
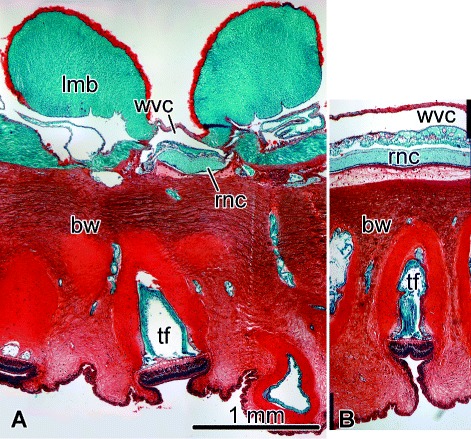
Organization of the uninjured radial nerve cord and surrounding tissues in the sea cucumber *H. glaberrima*. Paraffin sections stained with Safranin O, Fast Green and Weigert Iron Hematoxilyn. **A** Transverse section. **B** Longitudinal section. *bw*, body wall; *lmb*, longitudinal muscle band; *rnc*, radial nerve cord; *tf*, tube foot; *wvc*, water-vascular canal

The main components of the echinoderm CNS are five equidistantly spaced radial nerve cords (RNCs) (Fig. 1), each innervating the adjacent structures in the respective sector of the body. At the oral end of the animal, the five RNCs are joined together by a circumoral nerve ring. An established injury paradigm to investigate neural regeneration in echinoderms has been to completely cut one of the RNCs at about the mid-body level [1–3]. Among the most prominent components of the early post-injury response are a sharp increase in programmed cell death and extensive dedifferentiation of glial cells in the vicinity of the lesion. The dedifferentiated glial cells become highly proliferative and act as a major source of both new glial cells and new neurons in the regenerating segment of the radial nerve cord [1, 3]. The ability of the echinoderm glia to acquire pluripotency and take on a leading role in neural regeneration contrasts with the early post-traumatic events in mammals, where glial cells form a scar, which inhibits any subsequent regeneration across the wound gap [4, 5]. As the early processes in the lesioned CNS largely determine whether genuine regeneration or scarring will ensure, better understanding of the events that unfold shortly after injury in the echinoderm CNS may eventually lead to the development of better therapeutic strategies to stimulate neural regeneration in humans.

In an attempt to get insight into molecular mechanisms involved in control of cell activation and dedifferentiation in the regenerating echinoderm CNS, we investigated expression patterns of pluripotency factors in the RNC of a sea cucumber at various time points after injury [6, 7]. We identified 11 homologs of vertebrate pluripotency genes [8–10], of which only *Myc* expression showed significant increase in response to the RNC injury. The sea cucumber *Myc* is a homolog of mammalian Myc proteins, global transcription regulators that moderate expression of 10–15 % of the genome and play crucial roles in control of cell growth, proliferation, balance between self-renewal and differentiation, and apoptosis in various developmental contexts and during oncogenic transformation [11–14]. Interestingly, the expression level of the sea cucumber *Myc* was already elevated during the early response to RNC injury [7]. We therefore hypothesized that this increase in *Myc* expression was somehow associated with the initiation of CNS regeneration, however, the specific function(s) of the gene remained unknown.

In this study, we adapt RNA interference (RNAi)-mediated gene silencing to determine the roles played by *Myc* during the early post-injury response in the RNC of the brown rock sea cucumber *Holothuria glaberrima* Selenka, 1867. We show that elevated levels of *Myc* during the first two days after the injury are required for proper dedifferentiation of radial glial cells and for initiation of programmed cell death in the vicinity of the lesion. To our knowledge, the present study is the first implementation of RNAi methodology in regenerating adult echinoderms. The ability to use functional genomics tools makes it possible at last to experimentally decipher molecular pathways underlying post-traumatic organogenesis in these highly regenerating animals.

## Results

### Electroporation of DsiRNAs reduces Myc expression in the injured radial nerve

**Fig. 2:**
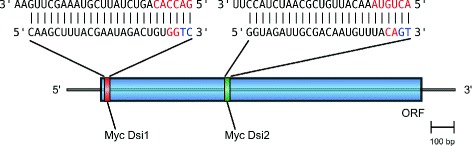
Diagram showing the sequences of the two *Myc*-targeting DsiRNAs (Myc Dsi1 and Myc Dsi2) and their target sites within the open reading frame (ORF) of the *H. glaberrima* *Myc* transcript. Red and blue letters indicate additional RNA and DNA bases, respectively, which distinguish DsiRNAs from classical 21-mer duplexes

**Fig. 3:**
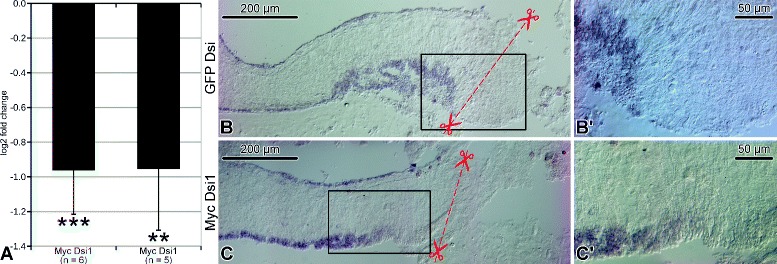
RNA interference-mediated *Myc* knockdown. **a** *Myc* expression in the regenerating radial nerve cord on day 2 after injury/DsiRNA injection as determined by qRT-PCR. Two DsiRNA constructs were used, Myc Dsi1 and Myc Dsi2, as described in Methods. Expression values are plotted as fold change relative to a negative control (a GFP-targeting DsiRNA) and expressed in a log2 scale. Error bars show standard deviation. ** *p*<0.01, *** *p*<0.001. **b-c**’ Representative in situ hybridization micrographs showing *Myc* expression in the radial nerve cord on day 2 after transection/DsiRNA injection. The upper (**b** and **b**’) and lower (**c** and **c**’) rows of micrographs show longitudinal sections of the RNC of an animal treated with a control (GFP-targeting) DsiRNA and an animal injected with one of the *Myc*-targeting DsiRNAs (Myc Dsi1), respectively. The micrographs on the right (**b**’ and **c**’) are high-magnification view of the boxed regions in the main micrographs on the left (**b** and **c**, respectively). The red dashed line indicates the position of the plane of injury. Note the absence of in situ hybridization signal from the cell bodies in the apical region of the ectoneural neuroepithelium of the RNC in the animal treated with Myc Dsi1, but not in the animal, which received the control DsiRNA injection

In order to determine the functional role of *Myc* in the early response to the CNS injury in *H. glaberrima*, we designed two different Dicer-substrate small interfering RNAs (DsiRNAs), designated as Myc Dsi1 and Myc Dsi2, which targeted distinct (non-overlapping) regions of the *Myc* transcript (Fig. 2). The decision to use longer DsiRNAs with a 25-nt sense strand and a 27-nt antisense strand rather than more traditional shorter 21-nt duplexes was based on the fact that DsiRNAs can be up to 100-fold more efficient than the classical 21-mers [15]. DsiRNAs were injected and electroporated into the RNC (Additional files 1 and 2). Besides reagent delivery, the injection procedure also served another function. The diameter of the injection needle was chosen to be greater than the width of the RNC, so that complete transection is achieved during the injection procedure (Additional file 2). Two days after surgery, quantitative real-time RT-PCR (qRT-PCR) showed that injection and electroporation of either Myc Dsi1 or Myc Dsi2 caused a significant ∼1.9-fold decrease in *Myc* mRNA expression compared with the injection of the control GFP-trageting DsiRNA (Fig. 3a), whereas the animals treated with the control DsiRNA themselves did not show any significant differences when compared with the animals injected with the vehicle alone.

Likewise, in situ hybridization shows that RNAi-mediated gene targeting causes *Myc* expression to fall below the detection limit in the cells adjacent to the site of injury/injection. No knockdown occurred when the animals were injected with the control GFP-targeting Dsi RNA (Fig. 3b-c’).

### Forced downregulation of Myc transcripts impairs dedifferentiation of radial glia

Glial dedifferentiation is one of the key cellular events that take place in the injured sea cucumber RNC shortly after transection [1, 3]. Under normal conditions, fully differentiated radial glial cells show typical palisade morphology, with their cell bodies mostly localized to the apical region of the neuroepithelium and the long basal processes extending through the entire thickness of the neural parenchyma [16, 17]. In response to injury, however, the glial cells loose their basal processes, which undergo fragmentation and become eventually phagocytosed by adjacent cells. The glial cells bodies persist in the apical region of the neuroepithelium and retain epithelial characteristics such as intercellular junctions and apicobasal cell polarity, but undergo extensive reogranization of the cytoskeleton and chromatin decondensation [1, 3].

**Fig. 4:**
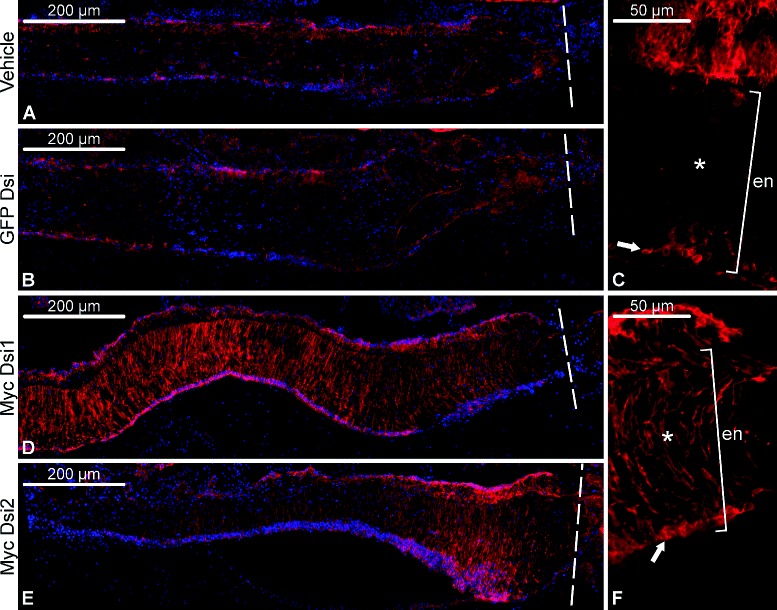
Representative micrographs showing the effect of *Myc* knockdown on glial dedifferentiation on day 2 post-injury/DsiRNA injection. The radial glial cells are visualized by immunostaining with the ERG1 monoclonal antibody (red) [17]; the nuclei (in **a, b, d, e**) were stained with Hoechst (blue). All micrographs are longitudinal sections with the plane of the injury *(dashed line)* to the right. **a** and **b** Control injections of the vehicle (**a**) and an irrelevant (GFP-targeting) DsiRNA (**b**). **c** Higher magnification of the radial nerve cord in a control (vehicle-injected) animal. **d** and **e** Injection of *Myc*-targeting DsiRNAs, Myc Dsi1 (**d**) and Myc Dsi2 (**e**). **f** Higher magnification of the radial nerve cord in a Myc Dsi2-injected animal. Note that in the control animals (**a-c**) the glial cells loose their long basal processes, while the cell bodies (*arrow* in **c**) remain in the apical region of the ectoneural neuroepithelium *(en)*. In contrast, many of the radial glial cells in the animals injected with *Myc*-targeting DsiRNAs (**d-f**) retained their basal processes, which extended through the underlying neural parenchyma *(asterisk)*

**Fig. 5:**
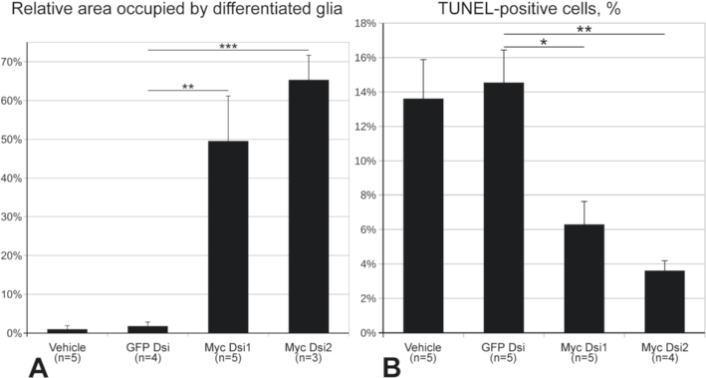
Effect of RNAi-mediated *Myc* silencing on glial dedifferentiation and programmed cell death. **a** Relative area occupied by fully differentiated radial glial cells within 1 mm from the wound in control animals injected with the vehicle or an irrelevant (GFP-targeting) DsiRNA (GFP Dsi) and in animals injected with *Myc*-targeting DsiRNAs (Myc Dsi1 and Myc Dsi2). Day 2 post-injury/DsiRNA injection. **b** Relative abundance of TUNEL-positive cells in the radial nerve cord within 1mm from the wound on day 2 post injury/DsiRNA injection. The control animals were injected either with the vehicle or with an irrelevant DsiRNA (GFP Dsi). The *Myc*-targeting DsiRNAs are designated as Myc Dsi1 and Myc Dsi2. The data are plotted as mean ± standard error. * *p*<0.05, ** *p*<0.01, *** *p*<0.001

In order to determine if there is any effect of RNAi-induced *Myc* knock-down on post-traumatic dedifferentiation of radial glial cells, we measured the relative area of the RNC occupied by glial cells, which still retained their basal processes (i.e., remained in the differentiated state) on day 2 post-injury. The measurements were performed on sagittal sections and covered the area spanning 1 mm from the site of the injury/injection. *Myc* knock-down had a highly significant effect on the extent of glial dedifferention (one-way ANOVA, *F*(3,13)=18.99,*p*=4.94×10^−5^). Electroporation of either of the two DsiRNAs, Myc Dsi1 or Myc Dsi2, resulted in a ∼50–60-fold increase in the size of the relative area of the radial nerve cord occupied by differentiated radial glia (with basal processes), whereas electroporation of the control (GFP-targeting) DsiRNA resulted in exactly the same phenotype as injection of the vehicle alone, i.e. extensive dedifferentiation (loss of the basal processes) in the radial glia (Figs. 4 and 5a). Therefore, *Myc* is required for induction of dedifferentiation of radial glial cells after CNS injury.

### Myc knock-down decreases the extent of programmed cell death in the injured radial nerve cord

Besides glial dedifferentiation, extensive programmed cell death is another characteristic feature of the early post-injury phase in the RNC [1, 3]. By day 2, the number of apoptotic cells in the vicinity of the injury increases 20-fold in comparison with uninjured animals and then starts to gradually return to the normal levels as regeneration progresses [3].

**Fig. 6:**
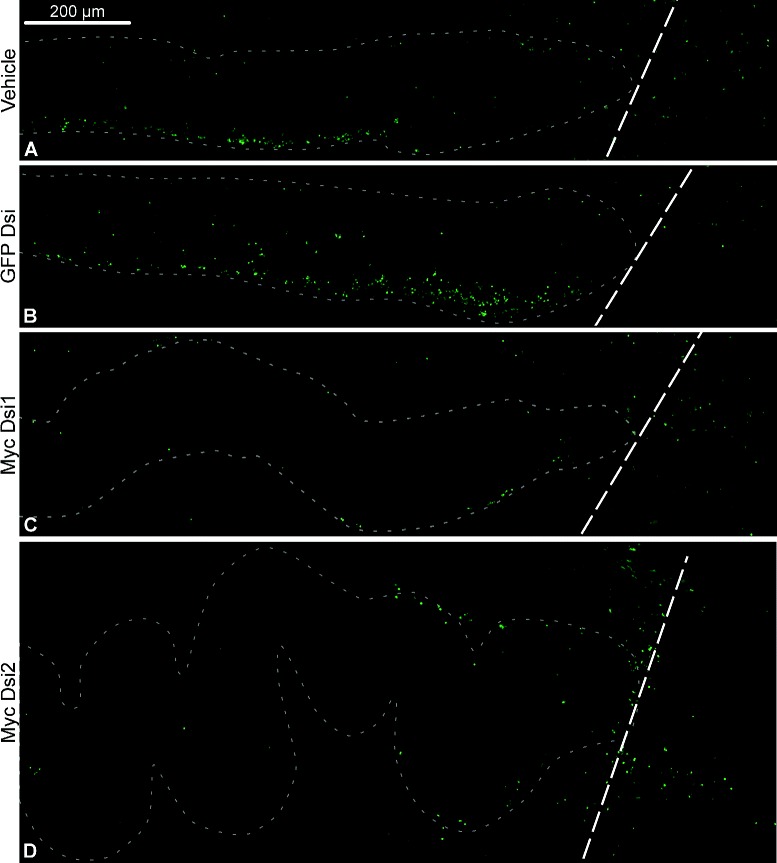
Representative micrographs showing the effect of *Myc* on programmed cell death on day 2 post-injury/DsiRNA injection. The cells undergoing programmed cell death were visualized with TUNEL assay (green). All micrographs are sagittal sections with the plane of the injury *(dashed line)* to the right. *Dotted line* indicates the outline of the radial nerve cord. **a** and **b** Control injections of the vehicle (**a**) and irrelevant control GFP-targeting DsiRNA (**b**). **c** and **d** Injection of *Myc*-targeting DsiRNAs, Myc Dsi1 (**c**) and Myc Dsi2 (**d**)

Given that Myc, depending on the context, is known either to trigger or suppress apoptosis in mammalian models [12, 18], we employed TUNEL (terminal deoxynucleotidyl transferase-mediated dUTP end labeling) assay to determine if *Myc* is involved in regulation of the programmed cell death in the injured radial nerve cord of *H. glaberrima*. There was a significant effect (one-way ANOVA, *F*(3,15)=9.4,*p*=9.7×10^−4^) of RNAi-mediated *Myc* down-regulation on relative abundance of TUNEL-positive cells in the vicinity (1 mm) of the wound on day 2 post-injury. *Myc*-targeting DsiRNAs caused a ∼2–4-fold decrease in the number of the cells undergoing programmed cell death, whereas the control GFP-targeting DsiRNA did not result in any changes in comparison with vehicle-injected animals (Figs. 5b and 6).

## Discussion

In our earlier experiments [6, 7], we identified *Myc* as the only pluripotency factor, whose expression significantly increased in response to CNS injury in the sea cucumber *H. glaberrima*. In vertebrates, Myc proteins are known to act as major hubs in gene regulatory networks, as they receive multiple inputs from various upstream signaling pathways and then in turn regulate expression of a wide range of downstream genes (up to 11–15 % of all promoters), including other transcription factors [12, 14, 19]. *H. glaberrimaMyc* might thus be a crucial component of the gene regulatory networks that trigger and control neural regeneration. It was therefore important to determine its specific functions.

Previously published evidence firmly established Myc as a regulator of cell differentiation. For example, c-Myc was one of the four components of the transcription factor cocktail that produced induced pluripotent stem cells from adult murine fibroblasts [8]. Myc downregulation, on the other hand, causes cells to exit the cell cycle and undergo differentiation [14]. Our data suggest that upregulation of Myc expression is also required for proper dedifferentiation of radial glial cells in the context of response to CNS injury in the sea cucumber *H. glaberrima*. Interestingly, this function of Myc is not unique to the rapidly regenerating echinoderm CNS, but can also be harnessed to improve recovery following neural injuries in animals with poor intrinsic regenerative abilities. Forced constitutive expression of v-Myc (the viral form of Myc) stabilized dedifferentiated state in rat embryonic radial glia. Upon transplantation into the injured adult spinal cord, these Myc-expressing cells were able to form bridges across the lesion and improved functional recovery [20]. The role of Myc in control of glial differentiation may, therefore, be part of a phylogenetically conserved program of neural regeneration throughout Deuterostomia.

Another well-known function of Myc is regulation of programmed cell death. Upregulation of Myc can either trigger apoptosis or protect cells from cell death, depending on the context, in which it is expressed [14, 18]. In sea cucumbers, a sharp increase in the extent of programmed cell death is one of the most notable components of the early reaction to the RNC injury [1, 3]. Since RNAi-induced Myc knock-down resulted in a significant decrease in abundance of apoptotic cells, we concluded that Myc positively regulates programmed cell death in the injured echinoderm CNS. It is not yet known, however, whether in this case the wave of cell death is merely a byproduct of the trauma or whether it is also required for initiation and coordination of the subsequent regeneration, as in head regeneration in *Hydra* [21] and tail regeneration in *Xenopus* tadpoles [22].

## Conclusions

Taken together, our data demonstrate for the first time the utility of RNA interference for determining functional roles of genes of interest in regenerating adult echinoderms. To our knowledge, *Myc* is the first transcription factor whose function in echinoderm regeneration was experimentally established. Quantitative PCR and in situ hybridization showed that injection and electroporation of Myc-targeting DsiRNAs resulted in a reliable knock-down of about 50 %. Analysis of the RNAi phenotypes revealed that in the context of post-traumatic neural regeneration in a sea cucumber *Myc* promotes acquisition of dedifferentiated state in glia and triggers programmed cell death. We hypothesize that Myc might be a component of a phylogenetically stable mechanism of deuterostome neural regeneration, which has even been preserved in a dormant state in poorly regenerating mammals. Further research into specific roles of regulatory genes associated with neural regeneration in echinoderms will improve our understanding of evolution of regeneration in deuterostomes and provide insights into which molecular pathways can be adjusted in mammals to improve their regenerative capacities.

## Methods

### RNA interference

Two different DsiRNA duplexes (Myc Dsi1 and Myc Dsi2) targeting different sequences in the coding region of the *H. glaberrimaMyc* transcript (GenBank KM281937) were designed using the siDirect (v2.0) software [23] and then manually edited following the guidelines of the IDT’s DsiRNA design manual [24]. Their sequences and target sites are shown in Fig. 2. The control DsiRNA was designed to target the green fluorescent protein (GFP) and had the following sequence: sense strand 5’–AGC UGA CCC UGA AGU UCA UCU GCA C–3’, anti-sense strand 5’–GUG CAG AUG AAC UUC AGG GUC AGC UUG–3’. Chemical synthesis of the DsiRNAs was outsourced to Integrated DNA technology, Inc., Coraville, IA.

The liophilized DsiRNA reagents (as received from the vendor) were resuspended in the RNase-free Duplex Buffer (IDT) to yield a 100 *μ*M solution, which was heated to 94 °C for 2 min and then slowly cooled down to room temperature to re-anneal the sense and anti-sense strands of the duplex. Fast Green (the tracking dye) was added to the solution to the final concentration of 0.01 %.

Adult individuals of *H. glaberrima* were induced to eviscerate by intracoelomic injection of 0.35M KCl. The animals were then anesthetized in 0.2 % chlorobutanol (Sigma) for 10–30 min. The inner (coelomic) surface of the body wall was exposed through the anus by pushing a glass rod against the epidermis, as described elsewhere [3, 6] (Additional file 1). Using a Hamilton syringe, we injected 11 *μ*l of a DsiRNA solution or the vehicle into the radial nerve cord. As the diameter of the needle was wider than the radial nerve cord, the latter was completely transected during the injection procedure. Next, a pin-and-paddle electrode (Nepa Gene, CUY661-3x7) was used for in vivo electroporation (Additional files 1 and 2). The pin anode was inserted into the canal made by the injection needle, whereas the paddle cathode was gently pressed against the longitudinal muscle band. Square electric pulses (5V, 50 ms) were generated by an ECM BTX 830 (Harvard Apparatus) electroporator and passed five times with the interval between pulses being 950 ms. The animals were then allowed to regenerate for two days before being analyzed.

The efficiency of our transfection (injection and electroporation) procedures was estimated in a separate experiment using tetramethylrhodamine-conjugated anionic dextran (Molecular Probes, D-3308). As above, 11 *μ*l of dextran solution (5 *μ*g/ *μ*l) was injected and electroporated into the radial nerve cord. Two days after injection, the tissues samples were fixed in 4 % paraformaldehyde in 0.01 M PBS (pH 7.4), cryosectioned, mounted in an antifading medium and analyzed using an epifluorescent microscope. Three animals were analyzed, and in all of them cells in large volumes of tissue extending at least 1,500 *μ*m from the point of injection incorporated the marker (Additional file 3). We therefore concluded that our transfection technique provided a reliable method of delivering RNA duplexes into cells of the adult sea cucumber radial nerve cords.

### qRT-PCR

Pieces of the radial nerve cord extending 4–5 mm on either side of the injury/injection (total length 8–10 mm) were excised with as little of the surrounding tissues as possible. Total RNA was extracted with Trizol reagent (Sigma). Real-time PCR was performed as previously described [6, 7]. The primers were designed to flank DsiRNA target sites, as recommended by Holmes et al. [25], and had the following sequences: 5’-CCA ACC GAG CAA TAA TGG CTA C-3’ and 5’-CGA ATG GTC CGA GAC TAA GGC-3’ (Myc Dsi1 site); 5’-CAA GAC TGT ATG TGG AGT GCT TTT C-3’ and 5’-AGT CCG AGG GTT GAC TAC AAT CAC-3’ (Myc Dsi2 site). Results were normalized relative to expression of *ATP6L* (the corresponding primers are 5’-CGG AGC AGG ACT TAG TGT CG-3’ and 5’-TTC CAA CAA ACA AAC GAG GCT-3’), which was previously identified among the most stably expressed genes in the normal and regenerating RNC of *H. glaberrima* [6]. Each condition was represented by cDNA samples from at least 5 different animals (biological replicates), and each sample was analyzed in two separate qRT-PCR reactions (technical replicates). Raw qRT-PCR data can be found in Additional file 4. Statistical processing of the data was performed using the MCMC.qpcr R package [26, 27] in the “classic“ mode, which allows a normalization procedure relative to control genes.

### In situ hybridization

Antisense riboprobes were transcribed from PCR-generated templates using Roche DIG-labeling mix. Tissue samples were fixed in 4 % paraformaldehyde overnight. Hybridization reactions were performed on 10 *μ*m-thick longitudinal cryosections as described earlier [28].

### Immunohistochemstry and TUNEL assay

Tissue samples were fixed and cryosectioned as above. At least three animals per condition were used. Immunostaining was carried out as described elsewhere [3, 17]. Briefly, after permeabilizing in 0.5 % Triton X-100 for 30 min, the sections were incubated in 0.1M glycine for 1 hour to quench residual autofluorescence and then blocked for 1 hour in 2 % normal goat serum. The primary ERG1 antibody [17] was applied overnight at 4 °C. After four washes with PBS (10 min each), the sections were incubated in the Cy3-conjugated goat anti-mouse secondary antibody (Jackson ImmunoResearch Laboratories, Inc) for 1 hour at room temperature. After washing off the excess antibody, the sections were mounted in an antifading medium.

Cells undergoing programmed cell death were identified using the Fluorescent FragEL™DNA Fragmentation Detection Kit (Calbiochem) as per the manufacturer’s protocol.

The sections were viewed and photographed with a Nikon Eclipse 600 microscope equipped with a SPOT RT3 camera (Diagnostic Instruments, Inc.). Cell counting and morphometric analysis were performed in at least three non-consecutive sections (at intervals of 30 *μ*m) per animal using the open source scientific image analysis software Fiji (http://fiji.sc/Fiji). The data were processed with the R statistical environment [27]. Differences between groups were analyzed with one-way ANOVA followed by Tukey’s post-hoc test.

## Additional files

Additional file 1
**Photographs illustrating different steps of the surgical procedure.** (1–3) Exposing the inner surface of the body wall through the cloacal opening. (4) Injection. (5, 6) Electroporation.

Additional file 2
**Diagram illustrating DsiRNA injection and electroporation procedure.** (A) Aqueous solution of DsiRNA (*blue*) was injected into the radial nerve cord (RNC) (*green*) with a Hamilton syringe. Since the diameter of the injection needle was greater than the width of the RNC, the injection procedure resulted in complete transection of the radial nerve. (B) The pin-and-paddle electrode was then used for electroporation. Note that the pin anode was inserted into the injection canal. *bw*, connective tissue of the body wall; *ce*, coelomic epithelium of the body wall; *ec*, epineural canal; *hc*, hypoeural canal; *rnc*, radial nerve cord; *wvc*, water-vascular canal.

Additional file 3
**Representative micrographs illustrating efficiency of the injection and electroporation technique.** To directly demonstrate the region of the radial nerve cord that is affected by our tranfection approach, we injected and electroporated aqueous solution of fluorescent dextran using the same technical parameters as for DsiRNA delivery. Red fluorescence signal shows extensive incorporation of the dye into numerous cells of the radial nerve. The region affected by electroporation extends more than 1 mm from the point of injection. (A) Low magnification view of the longitudinal section of the radial nerve cord. The dashed line indicates the injection needle canal. (A’) Higher magnification of the boxed area in (A). (A”) Detailed view of the boxed area in (A’).

Additional file 4
**Tabular (Excel spreadsheet) file containing raw qRT-PCR data.** The file includes columns of Ct values (one column per primer pair), as well as a two-column table of PCR efficiencies. The spreadsheets can be directly saved as comma-delimited (.csv) files and used as input to the MCMC.qpcr R package [26, 27].

Additional file 5
**Text file listing values of the relative area occupied by differentiated glial cells within 1 mm from the wound and the output of the corresponding R analysis.**

Additional file 6
**Text file listing values of the percentage of TUNEL labeled cells and the output of the corresponding R analysis.**

## Abbreviations

DsiRNA: Dicer-substrate small interfering RNA
GFP: Green fluorescent protein
RNAi: RNA interference
RNC: Radial nerve cord

## References

[1] Mashanov VS, Zueva OR, Heinzeller T (2008). Regeneration of the radial nerve cord in a holothurian: a promising new model system for studying post-traumatic recovery in the adult nervous system. Tissue Cell.

[2] San Miguel-Ruiz JE, García-Arrarás JE (2007). Common cellular events occur during wound healing and organ regeneration in the sea cucumber *Holothuria glaberrima*. BMC Dev Biol.

[3] Mashanov VS, Zueva OR, García-Arrarás JE (2013). Radial glial cells play a key role in echinoderm neural regeneration. BMC Biology.

[4] Karimi-Abdolrezaee S, Billakanti R (2012). Reactive astrogliosis after spinal cord injury-beneficial and detrimental effects. Mol Neurobiol.

[5] Cregg JM, DePaul MA, Filous AR, Lang BT, Tran A, Silver J (2014). Functional regeneration beyond the glial scar. Exp Neurol.

[6] Mashanov VS, Zueva OR, García-Arrarás JE (2014). Transcriptomic changes during regeneration of the central nervous system in an echinoderm. BMC Genomics.

[7] Mashanov VS, Zueva OR, García-Arrarás JE. Expression of pluripotency factors in echinoderm regeneration. Cell Tissue Res. 2014. in press doi:10.1007/s00441-014-2040-4. 10.1007/s00441-014-2040-4 PMC4323854 25468557

[8] Takahashi K, Yamanaka S (2006). Induction of pluripotent stem cells from mouse embryonic and adult fibroblast cultures by defined factors. Cell.

[9] Maki N, Suetsugu-Maki R, Tarui H, Agata K, Del Rio-Tsonis K, Tsonis PA (2009). Expression of stem cell pluripotency factors during regeneration in newts. Dev Dyn.

[10] Christen B, Robles V, Raya M, Paramonov I, Izpisúa Belmonte JC (2010). Regeneration and reprogramming compared. BMC Biol.

[11] Murphy MJ, Wilson A, Trumpp A (2005). More than just proliferation: Myc function in stem cells. Trends Cell Biol.

[12] Dang CV, O’Donnell KA, Zeller KI, Nguyen T, Osthus RC, Li F (2006). The c-myc target gene network. Semin Cancer Biol.

[13] Knoepfler PS (2008). Why myc? an unexpected ingredient in the stem cell cocktail. Cell Stem Cell.

[14] Meyer N, Penn LZ (2008). Reflecting on 25 years with MYC,. Nat Rev Cancer.

[15] Kim DH, Behlke MA, Rose SD, Chang MS, Choi S, Rossi JJ (2005). Synthetic dsRNA Dicer substrates enhance RNAi potency and efficacy. Nat Biotechnol.

[16] Mashanov V, Zueva O, Heinzeller T, Dolmatov I (2006). Ultrastructure of the circumoral nerve ring and the radial nerve cords in holothurians (Echinodermata). Zoomorphology.

[17] Mashanov VS, Zueva OR, Garcia-Arraras JE (2010). Organization of glial cells in the adult sea cucumber central nervous system. Glia.

[18] Gatti G, Maresca G, Natoli M, Florenzano F, Nicolin A, Felsani A (2009). MYC prevents apoptosis and enhances endoreduplication induced by paclitaxel. PLoS One.

[19] Fernandez PC, Frank SR, Wang L, Schroeder M, Liu S, Greene J (2003). Genomic targets of the human c-myc protein. Genes Dev.

[20] Hasegawa K, Chang YW, Li H, Berlin Y, Ikeda O, Kane-Goldsmith N (2005). Embryonic radial glia bridge spinal cord lesions and promote functional recovery following spinal cord injury. Exp Neurol.

[21] Chera S, Ghila L, Dobretz K, Wenger Y, Bauer C, Buzgariu W (2009). Apoptotic cells provide an unexpected source of Wnt3 signaling to drive *Hydra* head regeneration. Dev Cell.

[22] Tseng AS, Adams DS, Qiu D, Koustubhan P, Levin M (2007). Apoptosis is required during early stages of tail regeneration in *Xenopus laevis*. Dev Biol.

[23] Naito Y, Ui-Tei K (2012). siRNA design software for a target gene-specific RNA interference. Front Genet.

[24] Integrated DNA Technologies. Dicer Substrate RNAi Design. 2005. http://www.idtdna.com/pages/docs/default-source/technical-reports/DicerDesignRules.pdf?sfvrsn=1.

[25] Holmes K, Williams CM, Chapman EA, Cross MJ (2010). Detection of siRNA induced mRNA silencing by RT-qPCR: considerations for experimental design. BMC Res Notes.

[26] Matz MV, Wright RM, Scott JG (2013). No control genes required: Bayesian analysis of qRT-PCR data. PLoS One.

[27] R Core Team (2014). R: A Language and Environment for Statistical Computing.

[28] Mashanov VS, Zueva OR, García-Arrarás JE (2012). Posttraumatic regeneration involves differential expression of long terminal repeat (LTR) retrotransposons. Dev Dyn.

