# Supplementary figures and images for: Myc regulates programmed cell death and radial glia dedifferentiation after neural injury in an echinoderm
Source: BMC Dev Biol. 2015 May 30;15:24. doi: 10.1186/s12861-015-0071-z (PMC4448152; doi:10.1186/s12861-015-0071-z)

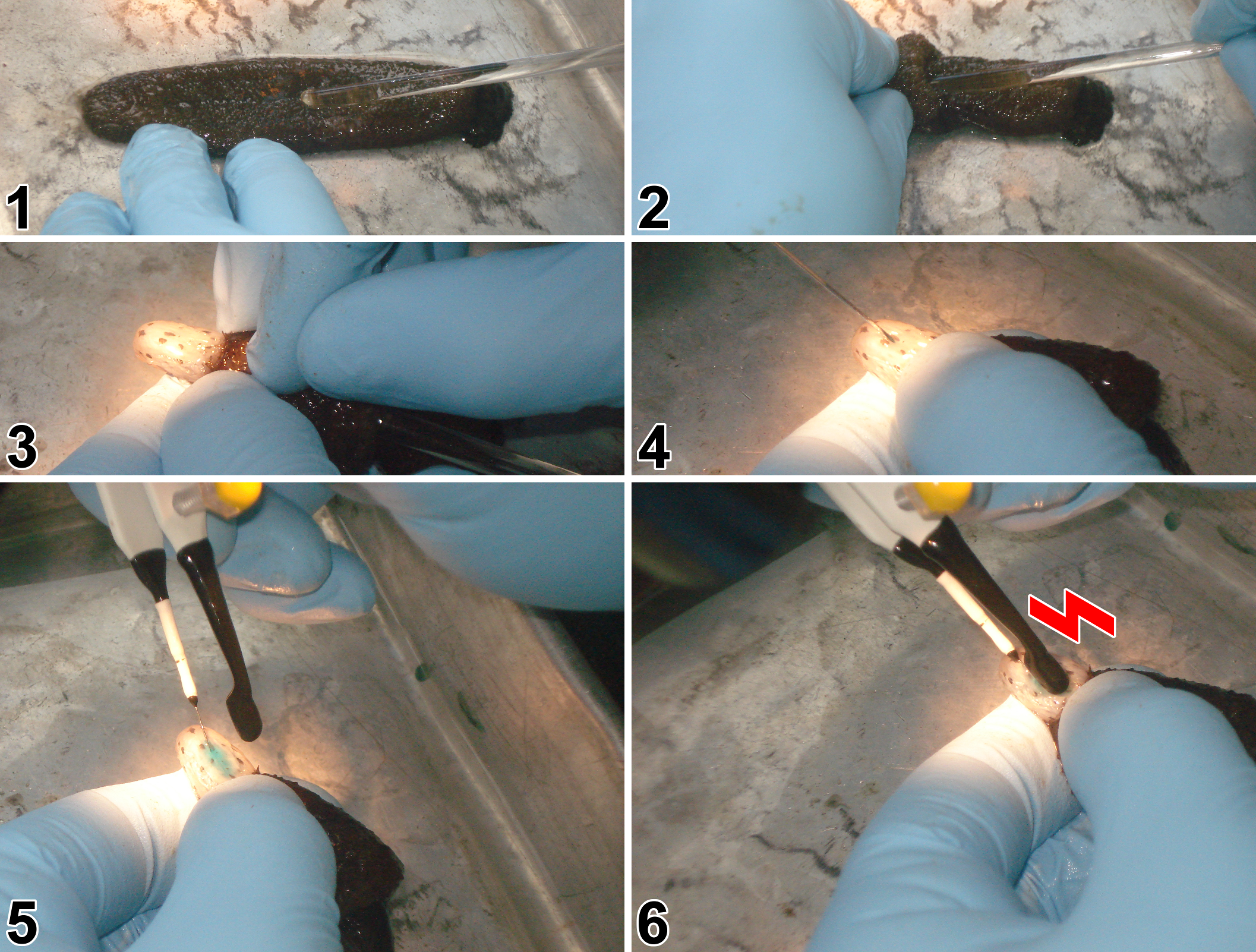

Supplement: Additional file 1 — Photographs illustrating different steps of the surgical procedure. (1–3) Exposing the inner surface of the body wall through the cloacal opening. (4) Injection. (5, 6) Electroporation. [file 12861_2015_71_MOESM1_ESM.tiff]

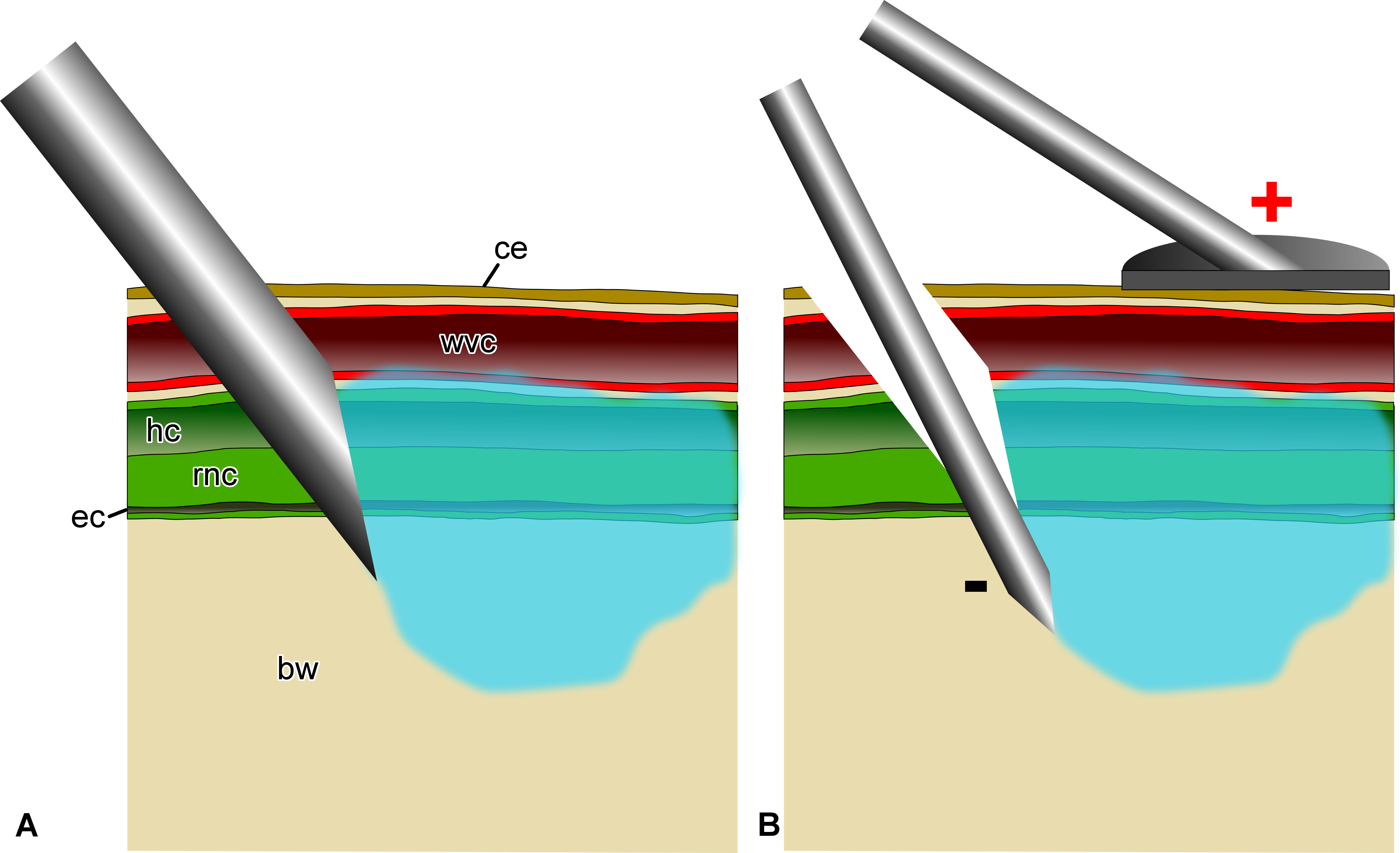

Supplement: Additional file 2 — Diagram illustrating DsiRNA injection and electroporation procedure. (A) Aqueous solution of DsiRNA (blue) was injected into the radial nerve cord (RNC) (green) with a Hamilton syringe. Since the diameter of the injection needle was greater than the width of the RNC, the injection procedure resulted in complete transection of the radial nerve. (B) The pin-and-paddle electrode was then used for electroporation. Note that the pin anode was inserted into the injection canal. bw, connective tissue of the body wall; ce, coelomic epithelium of the body wall; ec, epineural canal; hc, hypoeural canal; rnc, radial nerve cord; wvc, water-vascular canal. [file 12861_2015_71_MOESM2_ESM.tiff]

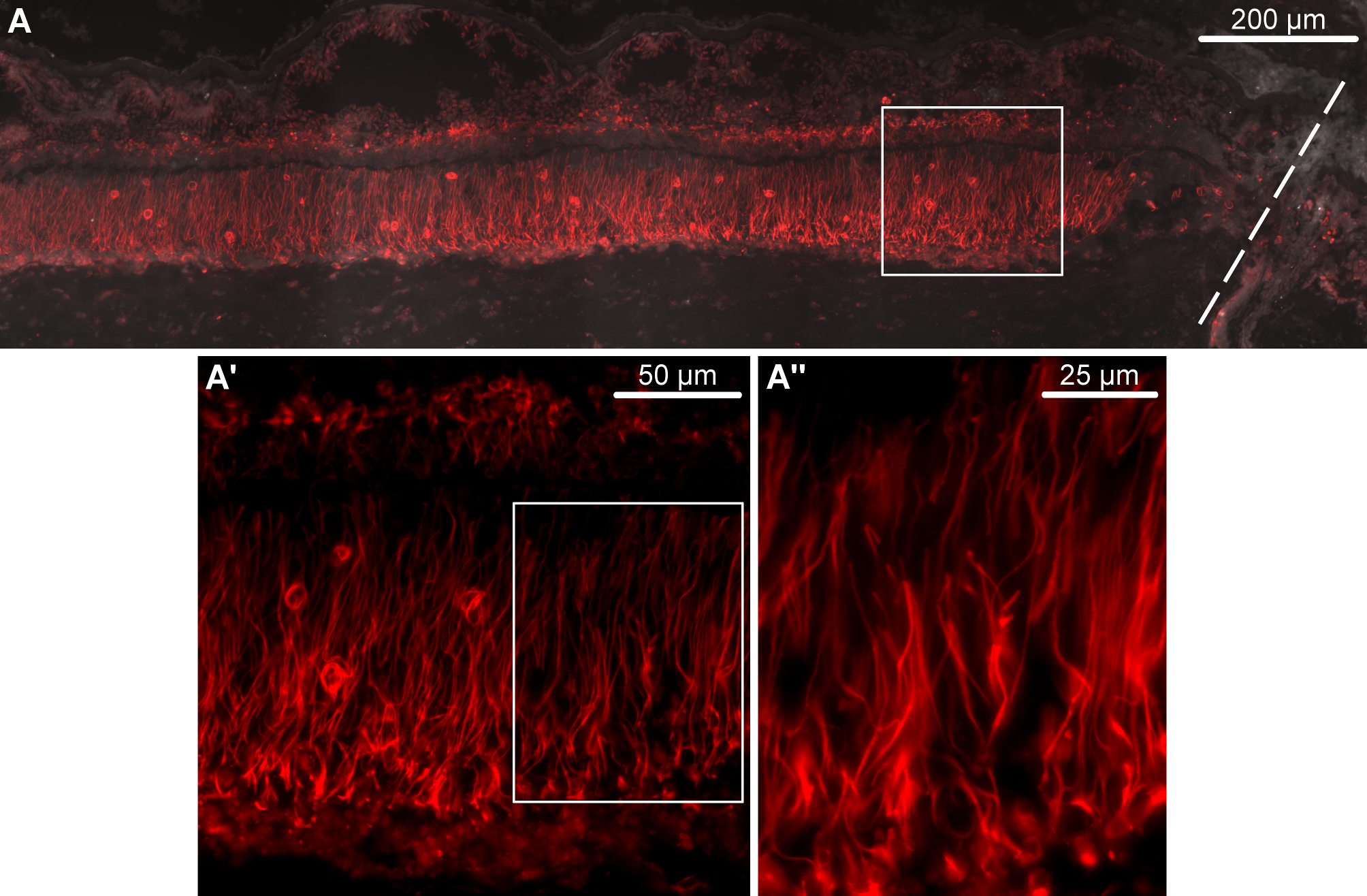

Supplement: Additional file 3 — Representative micrographs illustrating efficiency of the injection and electroporation technique. To directly demonstrate the region of the radial nerve cord that is affected by our tranfection approach, we injected and electroporated aqueous solution of fluorescent dextran using the same technical parameters as for DsiRNA delivery. Red fluorescence signal shows extensive incorporation of the dye into numerous cells of the radial nerve. The region affected by electroporation extends more than 1 mm from the point of injection. (A) Low magnification view of the longitudinal section of the radial nerve cord. The dashed line indicates the injection needle canal. (A’) Higher magnification of the boxed area in (A). (A”) Detailed view of the boxed area in (A’). [file 12861_2015_71_MOESM3_ESM.tiff]
